# RETRACTION: Effects of Dietary Waste Wet Dates (*Phoenix dactylifera*) on Growth, Feed Utilization, and Health Status of Nile Tilapia, *Oreochromis niloticus*, and Thin‐Lip Gray Mullet, *Liza ramada*, in a Polyculture System

**DOI:** 10.1155/anu/9805024

**Published:** 2026-07-15

**Authors:** Aquaculture Nutrition

RETRACTION: M.M. Toutou, A. Elkelish, and A.A. Soliman, “Effects of Dietary Waste Wet Dates (*Phoenix dactylifera*) on Growth, Feed Utilization, and Health Status of Nile Tilapia, *Oreochromis niloticus*, and Thin‐Lip Gray Mullet, *Liza ramada*, in a Polyculture System,” *Aquaculture Nutrition*, vol. 2025 (2025). https://doi.org/10.1155/anu/4684297.

The above article, published online on 17 December 2025 in Wiley Online Library (http://onlinelibrary.wiley.com), has been retracted by agreement between the Journal’s Editor‐in‐Chief, Antony J. Prabhu Philip; and John Wiley & Sons Ltd.

The retraction has been agreed following an investigation undertaken by the publisher, which identified concerns related to the data presented in the manuscript. More specifically, the validity of several data points shown in Tables 4–7 was brought into question and the underlying raw data were requested from the authors.

The authors provided the raw data, which were assessed by the publisher and the journal’s editorial board. Following an investigation, it was found that the raw data underpinning several means were identical for all replicates.

As a result of the investigation, the data and conclusions of this article are considered unreliable. Therefore, the article must be retracted.

The authors were informed of this retraction but did not provide a response.

